# Emergence of *Dirofilaria repens* (*Spirurida: Onchocercidae*) in dogs in Eastern Thailand

**DOI:** 10.14202/vetworld.2021.2851-2854

**Published:** 2021-11-06

**Authors:** Wanarit Jitsamai, Patchana Kamkong, Sariya Asawakarn, Piyanan Taweethavonsawat

**Affiliations:** 1 Parasitology Unit, Department of Veterinary Pathology, Faculty of Veterinary Science, Chulalongkorn University, Bangkok, Thailand; 2 Biomarkers in Animal Parasitology Research Group, Chulalongkorn University, Bangkok, Thailand; 3 Biochemistry Unit, Department of Veterinary Physiology, Faculty of Veterinary Science, Chulalongkorn University, Bangkok, 10330, Thailand

**Keywords:** *Dirofilaria repens*, dog, emerging, prevalence, Thailand, zoonosis

## Abstract

**Background and Aim:**

*Dirofilaria repens* is a zoonotic vector-borne parasite of dogs and cats. It is not commonly found in every part of Thailand, except the southern part. This study was conducted to investigate the prevalence of this parasite in Eastern Thailand in 2019.

**Materials and Methods:**

A total of 8003 blood samples were collected from private veterinary clinics and animal hospitals in Eastern Thailand. Blood parasites were examined using buffy coat thin blood smears with Wright-Giemsa staining. *D. repens* was morphologically identified and confirmed using the acid phosphatase activity technique.

**Results:**

The first case of *D. repens* was found in March 2019. The prevalence of *D. repens* from January to December 2019 was 0.44% (35/8003) (95% confidence interval 0.30-0.61).

**Conclusion:**

The prevalence data of *D. repens* in Eastern Thailand indicate that this parasitic infection should be considered as a zoonotic vector-borne disease. A strategic plan to control zoonotic transmission alongside a preventive program should be emphasized and encouraged among pet owners and veterinarians.

## Introduction

Several filarial nematodes are found worldwide, especially in tropical countries such as Thailand that have a suitable climate for the mosquito vector. In Thailand, filariasis is caused by *Dirofilaria immitis*, *Brugia malayi*, *Brugia pahangi*, and *Acanthocheilonema* (*Dipetalonema*) *reconditum* [1-4]. *Dirofilaria repens* is a nonpathogenic canine filarial nematode that is also found in cats and humans [5]. The epidemiology of *D. repens* was first investigated in Southern Europe and expanded to Northern Europe [6-8]. In recent years, some studies have suggested that *D. repens* in Asia has higher diversity than that in European countries, including Candidatus *D. hongkongensis* from Hong Kong and *D. repens* “Thailand II” found in Narathiwat Province, Thailand [5,9,10]. *D. repens* has also been detected in other parts of Thailand, including Bangkok, but the prevalence was very low [11]. Humans are considered as an accidental host for dirofilariasis. Humans infected with *D. repens* commonly manifest two clinical forms of filariasis, namely, subcutaneous and ocular [7]. Nodules in subcutaneous tissues are generally approximately 1 cm in size and sometimes may present clinical signs such as cutaneous larval migration, including irritation and itching [12]. The ocular form is primarily found in the subconjunctival region. In cases of chronic infection that is not diagnosed in a timely manner, the parasites can migrate into the peri-, intra-, and retro-ocular spaces, resulting in complications such as damaged vision, floaters, glaucoma, retinal detachment, vitreous opacity, loss of visual acuity, and blindness [13]. In Thailand, ocular dirofilariasis due to *D. repens* infection was reported to cause a cystic mass in the eyelid of a woman living in Phangnga province [14]. Recently, the first case of subconjunctival dirofilariasis was reported in a woman in Bangkok, Thailand [15].

The mode of transmission of *D. repens* is similar to that of *D. immitis*. Mosquitoes belonging to the genera *Anopheles*, *Aedes*, and *Culex* are potential vectors for *Dirofilaria* spp. [16,17]. *D. immitis* infection in dogs causes cardiopulmonary diseases, but *D. repens* infection causes milder disease in subcutaneous tissues [18]. Dirofilariasis caused by *D. repens* is considered as a nonpathogenic disease. However, recent research has suggested that subcutaneous dirofilariasis is associated with dermatological symptoms together with concomitant pruritus, neoplastic processes, inflammation, and blindness in dogs and humans [19]. It was observed that the blood parameters in dogs were altered during infection, with a reduction in the number of white blood cells, red blood cells, and platelets and an increase in alkaline phosphatase and creatinine activities [19].

In Thailand, the majority of studies have focused on the southern part of Thailand, which is an endemic area of filariasis as the climatic conditions are suitable for the vector. However, there are limited epidemiological data concerning canine subcutaneous ­dirofilariasis in eastern Thailand, which has a similar climate. Therefore, this study was conducted to explore the prevalence of filariasis in Eastern Thailand during 2019.

## Materials and Methods

### Ethical approval

The research protocol was approved by Chulalongkorn University Animal Care and Use Committee (approval no. 2031065).

### Study period and location

This retrospective study was conducted from January to December 2019. Records of *D. repens* were obtained from the central veterinary laboratory, Rayong branch, which collected canine blood samples from private veterinary clinics and animal hospitals in seven provinces in Eastern Thailand, including Chonburi, Rayong, Chanthaburi, Trat, Chachoengsao, Prachin Buri, and Sa Kaeo (excluding Pattaya city, a municipal area).

### Blood collection and parasite identification

A total of 8003 EDTA-blood samples were collected from owned dogs and submitted to the Central Veterinary Laboratory, Rayong branch. Buffy coat thin blood smears were prepared and stained with Wright-Giemsa. The stained smears were examined for the presence of microfilaria under a light microscope. Unsheathed microfilaria with two nuclei at the cephalic space was evaluated for their acid phosphatase activity to identify *D. repens* [20].

### Statistical analysis

The prevalence of *D. repens* is presented using descriptive statistics and 95% confidence intervals (CIs).

## Results and Discussion

**Figure-1:**
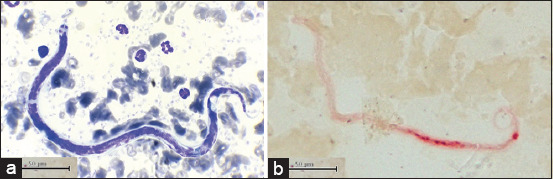
Microfilaria of *Dirofilaria repens* in (a) Giemsa-stained buffy coat smear and (b) acid phosphatase activity test.

**Figure-2:**
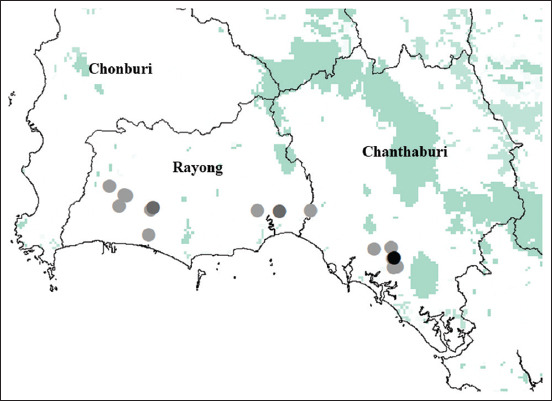
Locations of private veterinary clinics and animal hospitals and prevalence of filariasis demonstrated by dots (greyscale <2 cases, dark grey 2-4 cases, and black >4 cases) during January to December 2019. Source: The map was constructed using base map from GADM, version 1.0 for administrative areas and GLC2000 for land cover. (https://www.diva-gis.org/gdata)

From January to December 2019, a total of 8003 blood samples were examined, of which 0.44% (35/8003) (95% CI 0.30-0.61) were *D. repens*-positive. The microfilaria of *D. repens* was identified using the buffy coat smear with Wright-Giemsa staining and the acid phosphatase activity test (Figure-1a and b, respectively). A map of the private veterinary clinics and animal hospitals in Rayong and Chanthaburi Provinces is depicted in Figure-2. Coinfection with *B. pahangi*, *Hepatozoon canis*, *Babesia* spp., *Ehrlichia canis*, and *D. immitis* was observed (Table-1). The prevalence in each month is presented in Table-2; the highest prevalence of *D. repens* was observed in August, at 1.2% (95% CI 0.55-2.27).

**Table-1:** Coinfection of *D. repens* with other vector-borne pathogens.

Coinfection	Prevalence (n)	95% CI
Single infection of *D. repens*	0.32 (26)	0.21-0.48
*D. repens* with *D. immitis*	0.01 (1)	0.00-0.07
*D. repens* with *B. pahangi*	0.05 (4)	0.01-0.13
*D. repens* with *Babesia spp.*	0.01 (1)	0.00-0.07
*D. repens* with *B. pahangi* and *H. canis*	0.02 (2)	0.00-0.09
*D. repens* with *B. pahangi,* and *E. canis*	0.01 (1)	0.00-0.07

*D. repens=Dirofilaria repens, D. immitis=Dirofilaria immitis, B. pahangi=Brugia pahangi, H. canis=Hepatozoon canis, E. canis=Ehrlichia canis,* CI=Confidence interval

**Table-2:** Prevalence of *Dirofilaria* repens in each month from January to December 2019.

Month	Number of dogs	Prevalence (%)	95% CI
January	600	0.00	0.00-0.00
February	590	0.00	0.00-0.00
March	775	0.13	0.00-0.72
April	630	0.95	0.35-2.06
May	700	0	0.00-0.00
June	650	0.15	0.00-0.85
July	735	0.54	0.15-1.39
August	750	1.20	0.55-2.27
September	654	0.46	0.09-1.33
October	665	0.30	0.04-1.08
November	635	0.94	0.35-2.04
December	619	0.48	0.10-1.41

CI=Confidence interval

*D. repens* was first reported in the eastern part of Thailand in 2019. A large number of samples were collected from infected dogs, which may have influenced the prevalence recorded herein. However, we were not able to performed a gold standard test for microfilarial count such as the Knott or filtration test. This represents a limitation of this study and decreases the accuracy of our results in terms of sensitivity and specificity [21]. Furthermore, the samples were obtained from a clinical laboratory, and hence we do not have any details regarding the sex and breed of the infected dogs.

In the present study, we detected 35 *D. repens* cases among the 8003 examined samples in 2019, and three dogs showed clinical signs such as lethargy, loss of appetite, and epistaxis. It has been hypothesized that infected dogs do not present any clinical signs. Canine subcutaneous dirofilariasis is considered to be asymptomatic. However, some cases presented subcutaneous nodules. Circulating microfilaria causes cutaneous lesions, resulting in pruritus, alopecia, erythema, and diffused dermatitis [22]. In addition to the subcutaneous space, adult *D. repens* has been reported in several sites, including the bulbar subconjunctiva, retroperitoneal space of the pelvic cavity, and mesentery in the abdomen [23]. Although coinfection with *B. pahangi* was the most common in this study, the clinical presentations have not been previously reported. Natural coinfection of *D. repens* and *D. immitis* results in cachexia, with systemic muscle wasting. In a previous study, *D. immitis* and *D. repens* were found ectopic from cardiopulmonary and subcutaneous sites through histopathological investigation of the liver, kidney, and spleen, resulting in lymphocyte infiltration, fibrosis, and mineralization [23].

Human dirofilariasis due to *D. repens* infection commonly causes subcutaneous nodules. However, some cases have been reported in ectopic sites, including the lungs, scrotum, penis, spermatic cord, epididymis, and female mammary glands [24]. In ocular dirofilariasis, parasites were detected in periocular tissues [25]. To control zoonotic transmission, pet owners and veterinarians must be encouraged to follow a preventive program. Besides the aforementioned potential vector, *Armigeres* spp. is a potential vector in some regions [26,27]. The tropical fruit and rubber orchards that are abundant in the eastern agricultural area of Thailand serve as suitable habitats for *Armigeres* spp. as in the southern part, which is also rich in rubber orchards. The clinical signs of *D. repens* infection in humans and animals are mild and might be ignored [23], especially in orchard laborers and orchard guard dogs, for socioeconomic reasons.

## Conclusion

*D. repens* was first reported in eastern Thailand in March 2019 and was found to be most prevalent in August. *Armigeres* spp. is considered as a potential vector as there are suitable habitats in the fruit and rubber orchards that are commonly found in Eastern Thailand. Pet owners and veterinarians should be encouraged to follow a preventive and treatment program to manage zoonotic transmission.

## Authors’ Contributions

WJ and PT: Designed the study. WJ, PK, SA, and PT: Contributed to the analysis and interpretation of data. WJ and PT: Wrote the manuscript. PK and SA: Assisted in writing and revision of the manuscript. All authors have read and approved the final manuscript.
